# The first rare and fatal case of invasive aspergillosis of spinal cord due to *Aspergillus nidulans* in an Iranian child with chronic granulomatosis disease: review of literature

**DOI:** 10.18502/cmm.6.1.2551

**Published:** 2020

**Authors:** Mahin Tavakoli, Mohammad Taghi Hedayati, Hossein Mirhendi, Sadegh Nouripour-Sisakht, Newsha Hedayati, Fatemeh Saghafi, Setareh Mamishi

**Affiliations:** 1 Student Research Committee, Invasive Fungi Research Center, Mazandaran University of Medical Sciences, Sari, Iran; 2 Invasive Fungi Research Center, Mazandaran University of Medical Sciences, Sari, Iran; 3 Department of Medical Mycology and Parasitology, Isfahan University of Medical Sciences, Isfahan, Iran; 4 Cellular and Molecular Research Center, Yasuj University of Medical Sciences, Yasuj, Iran; 5 Student Research Committee, Invasive Fungi Research Center, Sari, Iran; 6 Department of Clinical Pharmacy, Faculty of Pharmacy, Shahid Sadoughi University of Medical Sciences, Yazd, Iran; 7 Department of Infectious Diseases, Children Medical Center, Tehran University of Medical Sciences, Tehran, Iran

**Keywords:** Aspergillus nidulans, Chronic granulomatosis disease, Invasive aspergillosis, Spinal cord

## Abstract

**Background and Purpose:**

Invasive aspergillosis (IA) of the central nervous system (CNS) is a devastating complication which is rarely reported in immunocompromised children.

In this case presentation, we reported a rare and fatal IA with spinal cord involvement in a 10-year-old child with X-linked chronic granulomatosis disease (CGD).

**Case report:**

The child had a previous history of pulmonary tuberculosis. A cervical spine X-ray revealed the involvement of cervical vertebrae (T4/T5) and* rib*s causing spinal cord compression and epidural abscess*.* The patient underwent a decompressive laminectomy and mass removal. The histopathology and culture results suggested IA. Despite the aggressive and prolonged therapy, he died within one year. *Aspergillus nidulans *was identified as the causative agent based on morphological and molecular studies.

**Conclusion:**

This synopsis represents the aggressive behavior of infection caused by *A. nidulans* in the CGD patient.

## Introduction

Chronic granulomatous disease (CGD) is a rare inherited disorder of phagocytic cells caused by defects in the nicotinamide adenine dinucleotide phosphate oxidase complex [1]. It may be diagnosed in childhood or adulthood; however, the majority of the affected patients are children under five years of age [2]. These patients may present with few to mild nonspecific clinical symptoms without fever or leukocytosis, even when seriously infected [1]. 

Although high levels of erythrocyte sedimentation rate (ESR) and serum C-reactive protein (CRP) may be the only indicators, serum CRP is more useful than ESR for the diagnosis and monitoring of infection in CGD patients [3]. The CGD patients may have concurrent bacterial and fungal infections [4]. In an attempt to identify patients with documented bacterial or fungal infections, the medical records of 268 patients with CGD were followed at a single center over 4 decades [4]. In the mentioned study, the incidence of fungal infections was restricted to *Aspergillus *species, with *Aspergillus fumigatus*, followed by* A. nidulans,* accounting for a higher proportion of IA [4].

*Aspergillus nidulans* is one of the most important and well-known species of the *Aspergillus* section *Nidulantes* [5]. Although *A. fumigatus* has been by far reported as the most common pathogen, *A. nidulans* is reportedly the most virulent pathogen disseminating to the adjacent bones and then to the brain, thereby resulting in mortality [4]. To date, diverse clinical cases caused by *A. nidulans* have been reported from patients with otomycosis, mycetoma, keratitis, sinusitis, pulmonary aspergilloma, and primary cutaneous infection associated with a Hickman catheter [6-14]. In addition, t*he proportion of *A. nidulans* infections* *has been reported to be significantly higher in CGD patients with osteomyelitis* [15, 16]. 

In the majority of cases, although CNS aspergillosis involves the brain, the damage to the spinal cord may be a very rare complication in which the spinal cord compression causes vertebral and tissue destruction [17, 18]. Spinal cord infection usually results from hematogenous dissemination from the lung; however, it can rarely develop through contiguous spread [17]. Herein, we report a fatal case of spinal aspergillosis due to *A. nidulans *following vertebral osteomyelitis in a child suffering from CGD and review all reported cases in the English literature. 

## Case report

The patient was a 10-year-old boy with confirmed CGD as an underlying disease at 11 months of age based on hematological and immunological tests (nitroblue-tetrazolium=0). He had several admissions to the Children Medical Center of Tehran, Iran. The patient had a previous history of recurrent episodes of pneumonia for which he was treated with broad-spectrum antibiotics and long-term prophylaxis, including itraconazole (5 mg/kg/day), along with co-trimoxazole (5 mg/kg/day) and IFN gamma (1.5 mcg/kg). At the age of nine years, the patient was hospitalized for several clinical signs and symptoms, such as knee pain, night sweets, lethargy, coryza, and progressive weakness. 

**Figure 1:**
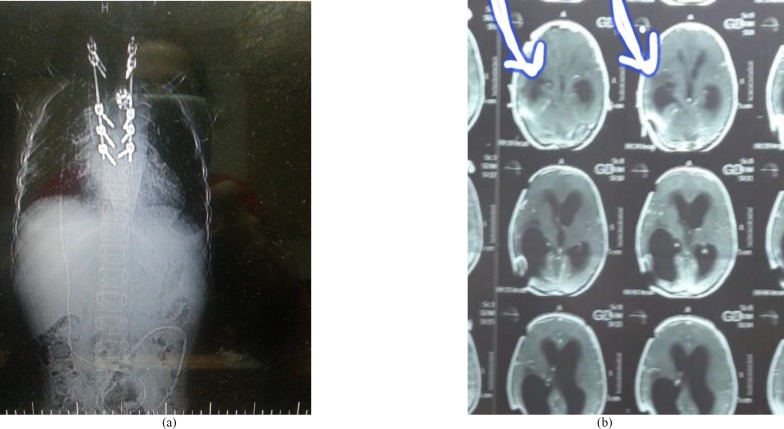
Imaging data in a chronic granulomatosis disease patient with invasive aspergillosis caused by *Aspergillus nidulans*; a) a plain X-ray revealing the laminectomy of L4-L5 with pedicular screw, along with post-operative changes, and exhibiting a destructive soft tissue density mass lesion centered on the body of T4-T5 vertebrae with adjacent prevertebral soft tissue formation mostly in the right side leading to central spinal column involvement and right fifth rib destruction, b) a computed tomography scan revealing brain abscess leading to hydrocephaly, showing extensive vasogenic edema in the right tempoparietal lobe, along with the dilations of the left ventricle and occipital horn of the right ventricle

**Figure 2:**
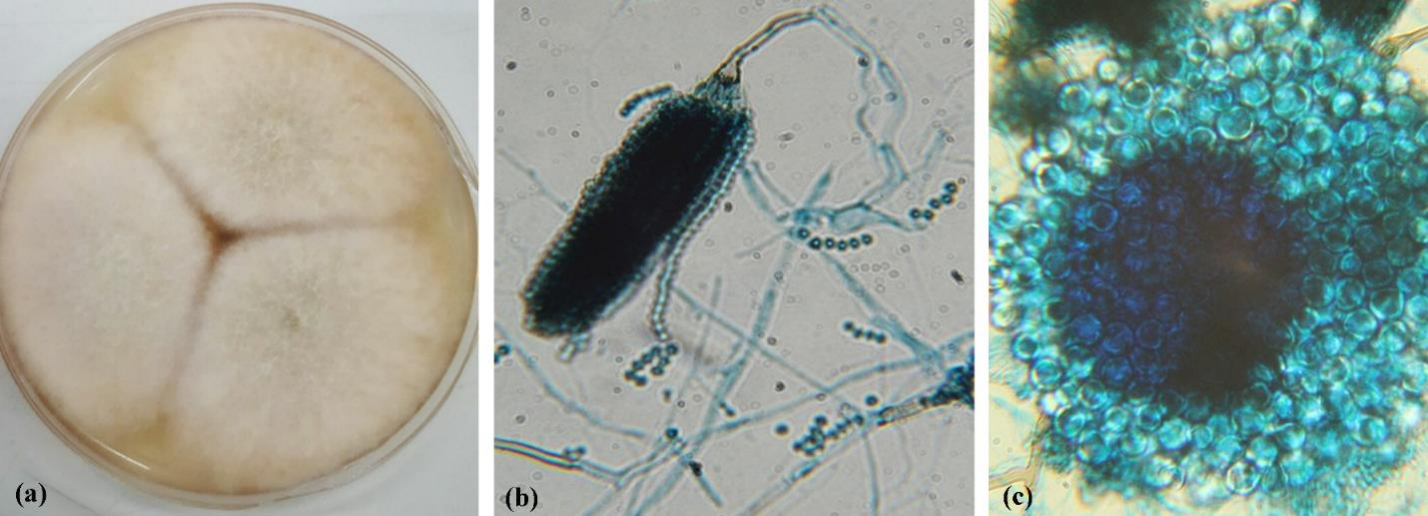
Microscopic image of *Aspergillus nidulans* isolated from the case; a) *A.* *nidulans* colonies on Czapek-Dox medium, b) Observation of conidial head structure of *A. nidulans* stained with Lactophenol Cotton Blue under light microscope (40X magnification), c) smooth round hull cell

A plain X-ray imaging showed a soft tissue density destructive lesion in T4-T5 vertebral bodies with adjacent prevertebral soft tissue mass involving the central spinal column. There was also evidence of an expansile lytic lesion involving the right fifth rib, laminectomy of L4-L5 with pedicular screw, along with post-operative changes (Figure 1). Laboratory findings included ESR of 75 mm/h, CRP of 69, white blood cell count of 6000/mm^3^, monocyte of 37.4%, hemoglobin of 7.8 g/dl, and thrombocyte of 383000/mm^3^. The results of histopathology and fungal culture obtained from surgical debridement samples revealed the diagnosis of invasive aspergillosis caused by *Aspergillus *species (Figure 2).

In addition, a microscopic examination showed abundant hull cells and the conidial head pattern of *A. nidulans* (Figure 2). The specific primer pair 5'-GGT AAC CAA ATC GGT GCT GCT TTC-3' and 5'-ACCCTC AGT GTA GTG ACC CTT GGC-3' was used to amplify β-tubulin genes and confirm the isolate as *A. nidulans* [19]. Then, the nucleotide sequence was deposited in GenBank under the accession number MK749954. At this time, the treatment was continued with amphotericin B deoxycholate (1 mg/kg/day) which was subsequently changed to voriconazole (9 mg/kg/day). Finally, caspofungin (50 µg/m^2^) was also added to the therapy, and the patient was discharged from the hospital. However, after a few months, this case was readmitted because of severe headache, blurred vision, and brain abscess, while receiving voriconazole therapy (200 mg/day). 

Treatment was continued with amphotericin B, caspofungin, voriconazole, and interferon-gamma. Due to the lack of antifungal response and deterioration of the symptoms, he underwent the insertion of a right parietal ventriculoperitoneal shunt. An urgent computed tomography scan of the brain did not depict any damage. However, it revealed the dilation of the left ventricle and dilation of the occipital horn of the right ventricle, along with extensive vasogenic edema in the right temporoparietal lobe (Figure 1). 

In spite of performing aggressive surgery and different treatments, the child passed away within one year. Postmortem antifungal susceptibility profile of the isolate was examined against itraconazole (Sigma-Aldrich Química, Madrid, Spain), amphotericin B (Sigma-Aldrich Química, Madrid, Spain), and voriconazole (Pfizer SA, Madrid, Spain) using EUCAST method [20]. The in vitro minimum inhibitory concentrations (MICs) of the mentioned antifungals were obtained as 1, > 16, and 0.25 mg/L, respectively. Furthermore, the minimum effective concentration for caspofungin (Merck & Co., Inc., Rahway, NJ) was estimated at 1 mg/L. This species was found to show resistance to amphotericin B (MIC>2 mg/L). 

## Discussion

The CGD is a rare disorder that prone the patients to bacterial and opportunistic fungal infections. [3]. Immunodeficiency, broad-spectrum antibiotic use, and *Aspergillus *colonization of a pre-existing pulmonary *cavity *may predispose the CGD patients to *Aspergillus* infection [21]. A review of data from the CGD registry revealed A. fumigatus* and *A. nidulans* as the most common species implicated in the lungs, bones, and brain [*16*]. The proportion of *A. nidulans* infections* *was significantly higher in patients with osteomyelitis, as well as in those receiving itraconazole prophylaxis [*16*]. Although *A. fumigates is the most frequent pathogen isolated from CGD patients, A. nidulans is more virulent and cause a higher fatality rate, compared to *A. fumigatus *[3]. 

The present study involved the report of a rare case of spinal aspergillosis following vertebral osteomyelitis in a child with CGD. In this case, a destructive soft tissue density mass lesion was manifested in the spinal column, reflecting the dissemination of infection from an adjacent organ, such as thoracic vertebrae and ribs. Aspergillus vertebral osteomyelitis is an infrequent infection, which can sometimes be complicated by spinal cord compression (Table 1). As indicated in Table 1, curative surgical intervention (laminectomy) is often attempted as a part of patient management for destructive lesions or spinal cord compression.

However, in this case, despite the implementation of aggressive and widespread therapies, brain abscesses were the late presentations of CNS manifestation which were mainly located in the cerebral cortex of the frontal, parietal, and occipital lobes. In a study performed by Van den Berg et al. [18], localized brain abscesses were reported in about 7% of patients with CGD, 38% of which was caused by *Aspergillus *species*.*

A literature search was performed using PubMed and Google Scholar databases. The search was accomplished using the following keywords: "Involvement of vertebral bodies" OR "Involvement of spinal cord" OR "CNS infection" AND "*A. nidulans*" AND "CGD patient". To date, including our case, only 19 relevant cases of vertebral osteomyelitis with or without the involvement of the spinal cord due to* A. nidulans* have been reported (Table 1). All patients were male with the age range of 2-21 years, with 75% being children and a mortality rate of 47.4%. Vertebral osteomyelitis was reported as the first manifestation in all patients in the presence or absence of pulmonary infection, often leading to the involvement of CNS and spinal cord by contagious dissemination. Therapeutic agents in these patients included liposomal amphotericin B, amphotericin B deoxycholate, flucytosine, itraconazole, and voriconazole, along with adjunctive therapy and aggressive surgery. 

Despite advances in early diagnosis and therapeutic approaches, CNS infection due to *A. nidulans* has still remained as a devastating opportunistic infection, often leading to death. In addition to the high virulence of this species, the use of amphotericin B, as the drug of choice, and the reduced susceptibility of *A. nidulans* to this antifungal, on the other hand, should be considered additional risk factors [22]. The use of gene therapy for the correction of underlying defects and control of invasive infections due to *Aspergillus* in CGD patients has also yielded a high success rate [23, 24].

**Table 1:** Demographic characteristics, clinical data, and treatment profiles of chronic granulomatosis disease patients affected with vertebral osteomyelitis and central nervous system aspergillosis due to *Aspergillus nidulans*

**Case**	**Study**	**Age/** **Sex**	**Predisposing factor**	**Involved site**	**Surgery**	**Pulmonary infection**	**Prophylaxis**	**Antifungal** **therapy**	**Adjunctive therapy**	**Brain abscess**	**Outcome**	
1	Redmond 1965 [32]	6/M	CGD	T1-T8 vertebrae, Ribs	Yes	Yes	No	AMB	No	No	Died	
2	Bujak et al., 1974 [33]	8/M	CGD	Fourth rib	No	Yes	Clindamycin	AMB	Leukocyte transfusion	No	Survived	
3	Altman et al., 1977 [34]	10/M	CGD	2nd–3rd ribs, Vertebrae	Yes	Yes	No	AMB	No	No	Survived	
4	White 1987 [35]	4/M	CGD	T8-10 Vertebrae, 7th ribs, Cord compression	Yes	Yes	No	AMB flucytosine, Itraconazole	Granulocyte transfusion	No	Survived	
5	Sponseller et al., 1991 [36]	4/M	CGD	8th-9th ribs, T6-L1 vertebrae	Yes	No	NA	AMB, imipenem	Granulocyte transfusion	No	Died	
6	Sponseller et al., 1991 [36]	9/M	CGD	4th rib	Yes	Yes	NA	AMB	No	No	Survived	
7	Sponseller et al., 1991 [36]	13/M	CGD	T3-T4 vertebrae, 4th rib	Yes	No	NA	AMB	No	No	Survived	
8	Kim et al., 1997 [37]	6/M	CGD	T5-T6 vertebrae	Yes	Yes	TMP-SMX	AMB, ITZ,	IFN-α, GM-CSF	No	Died	
9	Ozsashin et al., 1998 [38]	8/M	CGD	6th rib,	Yes	Yes	Itraconazole, TMP-SXT	Deoxycholate AMB, LAMB	IFNγ, G-CSF	No	Survived (BM transplantation)	
10	Segal et al., 1998 [7]	19/M	CGD	3rd rib, T3 Vertebrae, Spinal cord compression, temporal fossa involvement	Yes	Yes	No	AMB, itraconazole, flucytosine	IFNγ, Leukocyte transfusion, The instillation of granulocytes and hydrogen peroxide	Yes	Died	
11	Segal et al., 1998 [7]	16/M	CGD	3rd-4th rib, T4 vertebrae	Yes	Yes	No	Deoxycholate AMB, ketoconazole, itraconazole, liposomal AMB	IFNγ, leukocyte transfusion	No	Died	
12	Segal et al., 1998 [7]	4/M	CGD	Vertebrae, Cord compression	Yes	Yes	NA	AMB	Leukocyte transfusion	NA	Died	
13	Segal et al., 1998 [7]	7/M	CGD	Ribs, paraspinal mass, the involvement of sinuses, nasal cavity and orbits	Yes	Yes	No	Deoxycholate AMB, LAMB	IFNγ, leukocyte transfusion	Yes	Died	
14	Notheis et al., 2006 [23]	2/M	CGD	T2-T5 vertebrae, infiltration of spinal cord	Yes	Yes	TMP-SXT, Itraconazole	Deoxycholate AMB, voriconazole and caspofungin, posaconazole, LAMB,	leukocyte transfusion	No	Survived (ex-vivo gene therapy)	
15	Sastry et al., 2006 [39]	18/M	CGD	Rib, Vertebrae	Yes	Yes	TMP-SMX, Itraconazole	Liposomal AMB, voriconazole and caspofungin	No	Yes	Survived (BM transplantation)	
16	Dellapiane et al., 2008 [40]	21/M	CGD	T5-T7 vertebrae, SPINAL cord compression, ribs	Yes	Yes	TMP-SMX, ITZ	Liposomal AMB, caspofungin, voriconazole	IFNγ	probably	Died	
17	Bukhari et al., 2009 [41]	5/M	CGD	T1, T2, T4 and T6 vertebrae, Spinal cord compression	Yes	Yes	NA	Voriconazole	No	No	Survived	
18	Khalid 2018 [42]	9/M	CGD	3rd rib	No	Yes	TMP-SMX, ITZ	Voriconazole	No	No	Survived	
19	Present Case	10/M	CGD	T4-T5 vertebrae, fifth rib, spinal cord compression	Yes	Yes	ATT, Co-trimoxazole, Itraconazole	AMB, voriconazole and caspofungin	IFNγ	Yes	Died	


CGD: chronic granulomatosis disease, GM-CSF: granulocyte macrophage stimulating factor, TMP-SMX: trimethoprim-sulfamethoxazole, NA: non-available, ATT: anti-tuberculosis drugs, BMT: bone marrow transplantation

Briefly, our study highlights some important points. Firstly, the elevated levels of non-specific markers, such as ESR and CRP, may be helpful for the diagnosis and monitoring of vertebral osteomyelitis [25]. Nevertheless, significantly elevated levels of ESR and CRP are more valuable markers for the establishment of presumptive diagnosis and evaluation of patients by specific tests, such as galactomannan for IA [26]. Galactomannan as a predictive index could lead to the early diagnosis and rapid treatment of osteomyelitis due to aspergillosis [27]. Secondly, itraconazole is typically used for prophylaxis against IA. However, a long-term use of itraconazole can be a possible risk factor for developing IA due to *A. nidulans* in CGD patients [16]. 

Thirdly, in CGD patients suffering from IA, *IFN-γ* is crucial for both Th1-dependent protective immunity and phagocytic cell function against fungi [28]. Regarding this, IFN*-γ* was administered as adjunctive therapy in combination with voriconazole or amphotericin B in our patient, and a 10-fold increase of monocyte count was found without a significant difference in survival rate. 

Extracellular killing or *oxidase-dependent NETosis *induction is a possible reason for the difference of antimicrobial activity of CGD PMNs against *A. nidulans,* compared with *A. fumigates* [29]. Accordingly, despite the administration of higher doses of *IFN-γ to* CGD patients with *A. nidulans* osteomyelitis,* survival rate remains no change*. Moreover, in this patient, amphotericin B was considered first-line therapy; however, refractory to this antifungal was observed which can be the result of reduced susceptibility to amphotericin B [7]. Voriconazole has also shown higher response and improved survival rates than amphotericin B. This antifungal has been recommended as the preferred agent for the treatment of IA [30]. In addition, in clinical series, combination therapy with voriconazole and echinocandins has been employed to increase the efficacy of voriconazole monotherapy [31]. 

The present study involved the evaluation of the in vitro antifungal activity of only one isolate of *A. nidulans*. Therefore, the obtained results may not reflect the actual estimation of the clinical co-administration of voriconazole and echinocandins. 

## Conclusion

The determination of MIC based on the reference procedures is a gold standard to detect resistant clinical isolates and consider the best antifungal therapy to improve patient outcomes. Moreover, voriconazole is significantly more effective for the treatment of IA due to *A. nidulans* in the CGD patients, compared to amphotericin B deoxycholate; therefore, it should be preferred. 
